# Cefepime-Induced Neurotoxicity Can Be Confused With Neuroleptic Malignant Syndrome, Catatonia and Serotonin Syndrome: A Case Report

**DOI:** 10.7759/cureus.34223

**Published:** 2023-01-26

**Authors:** Mohammad Abu-Abaa, Dhay Bahadli, Osamah Abdulhussein, Ali Abdulsahib, Daniel Landau

**Affiliations:** 1 Internal Medicine Residency Program, Capital Health Regional Medical Center, Trenton, USA; 2 Internal Medicine, Capital Health Regional Medical Center, Trenton, USA; 3 Neurology, Capital Health Regional Medical Center, Trenton, USA

**Keywords:** altered mental status, neuroleptic malignant syndrome (nms), catatonia, serotonin syndrome (ss), cefepime induced neurotoxicity

## Abstract

Cefepime is a commonly used antibiotic. However, cefepime-induced neurotoxicity (CIN) is less commonly recognized. We describe a 75-year-old female on sertraline and risperidone who has been on cefepime for 28 days for treatment of osteomyelitis and presented with mutism, generalized rigidity, hyperreflexia, generalized stimulus-induced myoclonus, and reactive dilated pupils and found to have developed acute kidney injury. Although the diagnosis of serotonin syndrome (SS) and neuroleptic malignant syndrome (NMS) was suggested initially, the clinical picture was more compatible with CIN. Hemodialysis was suggested but gradual improvement in renal function allowed for gradual neurological recovery. This case highlights the importance of considering CIN in those who have been on Cefepime and present with altered mental status, especially in the appropriate clinical context and presence of risk factors. The overlap in clinical presentation between CIN, SS, NMS, and Catalonia may lead to a diagnostic challenge. Myoclonus seems to be characteristic of CIN and serves as a good clue to hint toward the diagnosis. This case helps to display the similarities and differences in the clinical presentation of these entities and therefore helps avoid confusion and prevents unnecessary therapeutic interventions.

## Introduction

Cefepime is a fourth-generation cephalosporin antibiotic. Its spectrum includes a wide range of Gram-positive and Gram-negative bacterium. It is a relatively safe antibiotic provided that its dose is adjusted according to eGFR [1]. Its main route of elimination is through the kidneys [1]. Its main route of elimination is through the kidneys [1]. The first report of cefepime-induced neurotoxicity (CIN) was in 1999 in a patient with end-stage renal disease (ESRD) on hemodialysis (HD) [2]. CIN usually presents with signs and symptoms of neuronal hyperexcitability including seizure, tremor, myoclonus, and altered mental status. Renal impairment is the main risk factor of CIN and leads to accumulation and increased cefepime crossing of the blood-brain barrier. The estimated CNS penetration of cefepime is 10%, which can be increased in case of renal impairment up to 45% and increase the half-life from two to 13 hours [3,4].

## Case presentation

A 75-year-old female patient presented to the emergency department (ED) with a few hours' history of altered mental status from the nursing home. She was noted to have a poor appetite for three days with progressive agitation followed by declining responsiveness. Past medical history is remarkable for dementia, hypertension, depression, hyperlipidemia, ischemic stroke, and left heel osteomyelitis on cefepime 1 gram every 8 hours. At the time of presentation, she had received 28 days of antibiotic treatment. Her home medications included mirtazapine, risperidone, sertraline, simvastatin, and lisinopril. Baseline mental status is alert and conversational. In ED, vital signs included a heart rate of 94 beats per minute, respiratory rate of 14 cycles per minute, a temperature of 36.5 degrees Celsius, and SpO_2_ of 96%. During the physical exam, she was spontaneously opening her eyes and staring but not responding to verbal stimuli and not following commands. The exam also revealed generalized hypertonia and hyperreflexia. Pupils were dilated and were reactive bilaterally. No evidence of clonus, ocular clonus, tremor, or diaphoresis was noted in the exam. Stimulus-induced myoclonus in all extremities was also evident in the exam. Complete blood picture and complete metabolic panel were significant for evidence of acute kidney injury (AKI) with increased serum creatinine at 6 mg/d (reference 0.5-1) from baseline of 0.8 mg/dL 20 days prior with estimated glomerular filtration rate (eGFR) below 10 ml/minute. Computed tomography (CT) head was unremarkable.

Dehydration was also evident clinically as well as by elevated blood urea nitrogen at 109 mg/dL (reference 7-17). No evidence of infection was noted on imaging tests, cultures, and other lab work. Creatine kinase (CK) level was 78 U/L (reference 30-135 U/L). The liver function test (LFT) was within normal limits. No significant fluctuation in blood pressure was noted. Initial differential diagnoses included neuroleptic malignant syndrome (NMS), serotonin syndrome (SS), cefepime-induced neurotoxicity (CIN), and catatonia. All antipsychotic medications were held along with cefepime. Antibiotic therapy was switched.

She was monitored with continuous electroencephalography (EEG). It showed generalized slowing with triphasic waves with no epileptiform discharges. Cefepime level was elevated at 38 mg/L (reference 5-10 mg/L). No hemodialysis was attempted, and no clinical seizure was evident. Recovery of renal function was slow over the course of three weeks. Ultimately, renal function improvement allowed for neurological recovery. Complete recovery was evident at a follow-up appointment one month later. She regained her full orientation and concentration with no longer clinically evident abnormal movements. Neurological examination showed normal reflexes and normal muscle tone.

## Discussion

Both NMS and SS are potentially life-threatening reactions to psychotropic medications. SS usually presents with altered mental status, hyperthermia, autonomic dysfunction, and neurological manifestations. NMS presents in a similar way but is also characterized by generalized hypertonia and muscle rigidity with abnormal hepatic function and evidence of rhabdomyolysis [5]. In this case, both NMS and SS were reasonable differentials as the patient was on antidopaminergic as well as anti-serotonergic medications. Generalized rigidity hinted toward NMS. However, her pupils were dilated, and she had hyperreflexia. Typically, hyporeflexia and normal pupils are observed in those with NMS [5]. Although hyperreflexia and dilated pupils are observed in SS, her pupils were reactive and generalized rigidity was not in keeping with SS. In addition, the patient did lack hyperpyrexia, CK, and LFT elevation. Catatonia was also a reasonable differential as the patient demonstrated immobility, mutism, staring, withdrawal and refusal to eat, and rigidity. However, generalized stimulus-induced myoclonus and hyperreflexia were more in keeping with cefepime-induced neurotoxicity (CIN) considering the clinical scenario and elevated serum cefepime level.

Cefepime is a relatively safe antibiotic and is the most common cephalosporin associated with neurotoxicity [6]. The exact incidence of cefepime-induced neurotoxicity is unknown, although it is estimated to be around 15% in intensive care unit (ICU) patients [7]. A prospective cohort study found the incidence of CIN among all patients receiving cefepime is at 1%, which increases renal impairment [8]. Guidelines exist in regard to dose modification in those with renal impairment. Renal impairment is the main risk factor for CIN [1]. The majority of cases were reported in elderly patients with a median of 69 years old and the majority 80% had renal impairment [7]. Although a cefepime blood threshold level of 22 mg/dL was suggested, pre-existing risk factors may lead to clinical neurotoxicity at a level below this threshold [7].

The mechanism, although largely unknown, is likely to involve gamma-aminobutyric acid (GABA) antagonism by both concentration-dependent competitions for GABA A receptors and reducing presynaptic GABA release and therefore neuronal hyperexcitability [1,7]. Other cephalosporins associated with neurotoxicity include first-generation like Cefazolin, second generation like Cefuroxime, and third generation like Ceftazidime [9].

Although widely variable, cefepime-induced neuronal hyperexcitability usually manifests clinically as tremor, seizure, myoclonus, aphasia, encephalopathy, drowsiness, stupor, and coma [7]. A seizure can be the only manifestation in 13% of cases. EEG typically shows a nonspecific triphasic wave pattern [10]. EEG may also show nonconvulsive status epilepticus (NCSE) in one-third of cases [11]. Altered mental status tends to be an early feature, while seizure and myoclonus are usually present later [7]. Myoclonus can be the only manifestation of CIN with or without minimal altered mental status [11]. Generalized rigidity has been reported in association with CIN [12,13]. Association with psychotic symptoms including catatonia and nihilistic delusion as in Cotard syndrome have also been suggested [14,15]. It is usually a clinical diagnosis of exclusion. Serum level only supports the diagnosis but does not preclude if it is normal.

In addition to renal impairment, other risk factors of CIN include older age, prior disruption of the blood-brain barrier including, central nervous system (CNS) infection, injury, sepsis, and uremia. Hypoalbuminemia is also a risk factor for reducing the number of bound drugs [16]. In our case, risk factors that contributed to CIN in addition to renal impairment included prior brain damage secondary to ischemic stroke and dementia as well as older age. Although dose adjustment is likely to help prevent neurotoxicity, it is reported that around 25% of cases happen despite appropriate dosing [2]. Symptoms usually start within four days of initiation of cefepime and are expected to resolve within three days after cefepime discontinuation, which is the only definitive treatment [2]. Antiepileptic medications are usually not needed except in case of clinical seizure. Hemodialysis can be used in case of an emergency need to reverse CIN.

## Conclusions

CIN should always be suspected in those with altered mental status and renal impairment who have been on cefepime therapy. CIN shares clinical features with SS, NMS and catatonia which can lead to clinical confusion in the appropriate clinical scenario. Generalized rigidity is commonly seen and can be mistaken for NMS. Hyperreflexia and dilated pupils can be seen and confused with SS. However, in CIN the pupils are reactive and the patient lacks hyperpyrexia, CK and LFT elevation. Myoclonus is a late and an important feature to help distinguish CIN and can be the only presenting feature. CIN can also present with features of catatonia like mutism, staring, withdrawal and refusal to eat as well as psychotic features. Cefepime use should always be doe-adjusted based on renal function and the likelihood of CIN should be foreseen and patients should be closely followed.
